# Association between cytomegalovirus end-organ diseases and moderate-to-severe dementia: a population-based cohort study

**DOI:** 10.1186/s12883-020-01776-3

**Published:** 2020-05-28

**Authors:** Kyoung Hwa Lee, Da Eun Kwon, Kyung Do Han, Yeonju La, Sang Hoon Han

**Affiliations:** 1 Division of Infectious Disease, Department of Internal Medicine, Yonsei University College of Medicine, 211 Eonju-ro, Gangnam-gu, Seoul, 06273 Republic of Korea; 2 Department of Biostatistics, College of Medicine, The Catholic University of Korea, Seoul, Republic of Korea

**Keywords:** Alzheimer’s disease, Cytomegalovirus, Claims data analysis, Dementia, End-organ diseases, South Korea, Vascular dementia

## Abstract

**Background:**

The association between cytomegalovirus (CMV) and dementia remains controversial. Previous studies have suggested that CMV serostatus, as assessed by serum immunoglobulin G, plays a role in neurodegeneration with cognitive impairment. We aimed to evaluate the association between CMV tissue-invasive end-organ diseases and moderate-to-severe dementia.

**Methods:**

The ICD 10th revision codes from the National Health Insurance Database covering the entire population of the Republic of Korea were used to classify patients into exposed (*n* = 687, age ≥ 40 years, with CMV disease) and unexposed (*n* = 3435, without CMV disease) groups, matched by age and sex at a 1:5 ratio of exposed: unexposed. All non-HIV-1-infected subjects selected during 2010–2014 with a washout period of the previous 4 years were followed up until December 2016 to identify newly diagnosed cases of moderate-to-severe dementia.

**Results:**

Multivariate regression model (M3) adjusted for age, sex, low income, body mass index, transplantation status, malignant neoplasms, end-stage renal disease on dialysis, type 2 diabetes mellitus, hypertension, and dyslipidaemia showed a significantly higher incidence of dementia (odds ratio: 1.9; 95% confidence interval: 1.2–2.8) in the exposed group than that in the unexposed group. The risk of vascular dementia (2.9, 1.1–7.5) was higher than that of Alzheimer’s disease (1.6, 1.0–2.6) in the exposed group in M3. In M3, patients aged 40–59 years with CMV diseases had a significantly higher risk of all kinds of dementia than those aged 60–79 and ≥ 80 years (11.7, 2.5–49.4 vs. 1.8, 1.1–3.2 vs. 1.3, 0.5–2.8; *P* = 0.025).

**Conclusions:**

CMV diseases may be associated with the risk of moderate-to-severe dementia.

## Background

Dementia is a neurologic disease defined as a gradual decline in memory and cognitive function [1]. The worldwide dementia burden, estimated to be 47 million cases in 2015, is expected to double every 20 years [1, 2]. Although the aetiology and pathogenesis of dementia remain unclear, it could be caused by brain damage due to vascular ischaemia, and/or hereditary, sporadic, or age-related factors [2, 3]. In addition, the hypothesis that infection may be a risk factor for Alzheimer’s disease (AD) has been supported by neuroinflammation and disease pathology [4, 5]. In vascular dementia (VaD), the second most common cause of dementia, neurogenic inflammation due to vascular damage could develop into cognitive dysfunction [6, 7].

Past ubiquitous human cytomegalovirus (CMV) infection can present in several forms, from life-long asymptomatic latency to tissue-invasive end-organ diseases. Because CMV is associated with several chronic inflammatory diseases, particularly vascular disorders and immunodysfunction, it has been suggested that there is also an association between CMV and dementia [7–9]. CMV deoxyribonucleic acid (DNA) was more dominant in the brain tissue of subjects with VaD than in those without VaD [8]. In addition, CMV was associated with AD development and declining global cognition [9, 10]. However, most studies on CMV and dementia are based on seroprevalence or the serostatus of latent CMV and were assessed using serum CMV immunoglobulin G (IgG). Although serum CMV IgG was suggested to play a role in neurodegeneration with cognitive impairment [7, 9, 10], a significant association between CMV serostatus indicating past infection without virus replication and dementia was not observed in a recent long-term follow-up study and meta-analysis [11, 12].

The Republic of Korea has high rates of CMV seropositivity [13]. Moreover, our country strictly applies the registration of CMV tissue-invasive end-organ diseases and moderate-to-severe dementia using the national health insurance system [14, 15]. Therefore, confirming an association between CMV tissue-invasive end-organ diseases that present with active virus replication and severe tissue inflammation through reactivation of primary or past latent infection and moderate-to-severe dementia with cognitive impairment enough to interfere with social and occupational functioning and daily living could provide clearer evidence of CMV causality in dementia than previous studies that were based on known seroprevalence. This study aimed to evaluate the association between CMV tissue-invasive end-organ diseases and moderate-to-severe dementia.

## Methods

### Extraction of nationwide population data

In the Republic of Korea, the Korean National Health Insurance Service (NHIS) requires mandatory registration for the entire population. These compulsory subscribers pay for health insurance based on their income level [16]. All medical institutions present claims on diseases diagnosed using the International Statistical Classification of Diseases and Related Health Problems 10th Revision (ICD-10) codes, World Health Organization (WHO) [16, 17]. Rare incurable diseases (RIDs) including human immunodeficiency virus-1 (HIV-1) infection, CMV end-organ diseases, cancer, and moderate-to-severe dementia are controlled by the Korean NHIS [14–16, 18]. Patients with RIDs receive significant health coverage by paying 5% of their healthcare costs. Therefore, the process of diagnosing RIDs and receiving medical claims should reflect accurate data [14, 16, 18]. The Korean Health Insurance Review and Assessment (HIRA) plays an important role in inspecting this process. We used a medical dataset extracted from the National Health Insurance Database (NHID), which was submitted to the HIRA. The NHID includes big data comprising 1.3 trillion records with information regarding medical diagnosis, treatment results, long-term care insurance for elderly patients, registration information on RIDs, and the status of medical institutions [14, 18]. This study was approved by the Institutional Review Board of the Gangnam Severance Hospital, Yonsei University College of Medicine. A waiver of informed consent and relevant permission forms were obtained from the National Health Insurance Sharing Service.

### Study population and design

From January 2010 to December 2014, the data from 1557 enrolled patients diagnosed with CMV tissue-invasive end-organ diseases and RID were extracted from the NHID. A 4-year washout period was considered (between January 2006 and December 2009) to evaluate the effect of CMV disease on new-onset moderate-to-severe dementia. Based on unique RID codes linked to ICD-10 codes at retrospective enrolment, we did not find any subjects with moderate-to-severe dementia in either group. Twenty-one patients diagnosed with moderate-to-severe dementia during the washout period and twenty-four HIV-1-infected individuals were excluded. Among the remaining 1512 patients, 687 with CMV tissue-invasive diseases aged ≥40 years were selected for the group of exposed individuals with CMV diseases. For the case-control cohort study, 3435 age- and sex-matched individuals were selected for the unexposed with no CMV diseases group at a 1:5 ratio. Our study design was not a retrospective matched-pairs analysis using a propensity score model. Participants from both groups were followed up until December 2016. The incidence rate (IR) per 1000 person-years was calculated by dividing the number of events (new-onset of moderate-to-severe dementia) with the follow-up duration of participants. The person-years in participants without an event of new-onset dementia was calculated from the follow-up time between enrolment and the date of follow-up end or death.

### Definition

A diagnosis of CMV tissue-invasive end-organ diseases can be established if the following are observed: (1) histopathologic features, including the presence of inclusion bodies and a positive finding in immunohistochemical staining for CMV; or (2) detection of CMV itself or its DNA in body fluids or tissue using the pp65 antigen measurement, culture, or nucleic acid amplification test [19, 20]. Moderate-to-severe dementia was diagnosed only by neurologists or psychiatrists according to the essential diagnostic methods and criteria by the Korean NHIS: (1) abnormal brain imaging finding (computed tomography or magnetic resonance imaging or fluorodeoxyglucose-positron emission tomography); and (2) ≥ two points in clinical dementia rating or ≥ five points in global deterioration scale and ≤ 18 points in mini-mental status examination; and/or (3) abnormal neuropsychological test (Seoul neurophysiological screening battery or consortium to establish a registry for Alzheimer’s disease assessment pocket or literacy-independent cognitive assessment) [21, 22].

CMV diseases are given a unique V104 code for RID registration. The V104 code is consistent with the specific B25 codes in the ICD-10, including those for all types of CMV tissue-invasive end-organ diseases, such as cytomegaloviral pneumonitis (B25.0), cytomegaloviral hepatitis (B25.1), cytomegaloviral pancreatitis (B25.2), other cytomegaloviral diseases (B25.8), and cytomegaloviral diseases, unspecified (B25.9) [17]. This did not include the codes for congenital CMV infection (P35.1) or cytomegaloviral mononucleosis (B27.1) [14, 17]. Furthermore, a dementia diagnosis for RID registration using a unique V800 and V810 code was consistent with the ICD-10 codes: AD (early or presenile onset or type 2 [F00.0/G30.0], late or senile onset or type 1 [F00.1/G30.1], atypical or mixed type [F00.2/G30.8], and unspecified [F00.9/G30.9]), VaD (acute onset [F01.0], multi-infarct or predominantly cortical dementia [F01.1], subcortical [F01.2], mixed cortical and subcortical [F01.3], and other or unspecified [F01.8 or F01.9]), and other dementia (dementia in Pick disease [F02.0/G31.0], in Creutzfeldt-Jakob disease [F02.1/A81.0], in Huntington disease [F02.2/G10], dementia in Parkinson disease [F02.3/G20], with Lewy bodies disease [F02.8/G31.8] and frontotemporal dementia [G31.0]) (Supplementary Table 1) [17, 23, 24].

The solid organ transplantation (SOT) recipients had V005 (kidney), V013 (liver), V015 (heart), and/or V277 (lung) codes according to the transplant organ for RID registration, which are consistent with the Z94 codes in the ICD-10 (Z94.0, Z94.1, Z94.2, Z94.3, and Z94.4 for the kidney, heart, lung, heart and lung, and liver, respectively). End-stage renal disease (ESRD) on dialysis was identified using V001 and V003 RID codes, identical to ICD-10 N18.5 code (chronic kidney disease, stage 5). HIV-1-infected individuals were identified using the V103 RID code in compliance with ICD-10 B20-B24 codes [17]. In addition, hypertension (I10-I13, I15), type 2 non-insulin-dependent diabetes mellitus (NIDDM) (E11), dyslipidaemia (E78), malignant neoplasms including haematologic malignancies and excluding in situ neoplasms (C00-C86.6, C88, C90-C97, and D37–48), and haematopoietic stem cell transplantation (HSCT) recipients (Z94.8) were identified using ICD-10 codes [17].

Body mass index (BMI) was calculated as weight/(height X height) (kg/m^2^) and categorised as < 25 and ≥ 25 kg/m^2^. Low income status was defined as the lower 25th percentile of annual household income, based on data from the 2010 South Korean Population and Housing Census [14].

### Statistical analysis

Categorical and continuous data were presented as numbers (percent) and mean ± standard deviation, respectively. The exposed and unexposed groups were compared using the *χ*^2^ and independent t-tests. Kaplan-Meier curves adjusted for age and sex were used to analyse the incidence and probability of dementia according to the presence of CMV diseases. In survival analyses, the time to diagnosis was determined as events indicating diagnostic time, to the time of new-onset moderate-to-severe dementia. Censored data were determined as death or follow-up end prior to the event, that mainly occurred owing to late enrolment in the cohort. Our study did not have type 2 censoring by loss to follow-up or drop out. The proportion of censored data in the exposed and unexposed group was similar (1.18-fold, 14.6% vs. 12.4%, respectively). Multivariate logistic regression analyses using model 1 (M1, non-adjusted), model 2 (M2, adjusted for age, sex, low income status, and BMI), and model 3 (M3, adjusted for age, sex, low income status, BMI, SOT and/or HSCT recipients, malignant neoplasms, ESRD on dialysis, NIDDM, hypertension, and dyslipidaemia) were performed to evaluate the impact of CMV diseases on moderate-to-severe dementia development. Statistical analyses were performed using the Statistical Analysis System (SAS) program (version 9.2; SAS Institute, Cary, NC). Two-tailed *P* values< 0.05 were considered significant.

## Results

### Clinical characteristics according to CMV diseases

The mean age (percentage of subjects aged ≥60 years) and percentage of males in the study cohort was 58 years (40.9%) and 56.8%, respectively. The follow-up duration was significantly longer in the unexposed group (4.1 ± 1.6 vs. 3.7 ± 1.8 years, *P* <  0.001). BMI was similar between the two groups; however, the exposed group had significantly higher rates of NIDDM (29.8% vs. 11.2%), hypertension (47.7% vs. 28.4%), and dyslipidaemia (29.3% vs. 17.7%) (*P* <  0.001). In addition, the exposed groups had significantly higher percentages of co-morbid conditions, including SOT or HSCT status, malignant neoplasms, and ESRD on dialysis (all conditions *P* <  0.001). The incidence of all moderate-to-severe dementia types was significantly higher in the exposed group (5.5% vs. 3.1%, *P* = 0.001). The incidence of VaD (1.0% vs. 0.4%, *P* = 0.018) but not AD (3.4% vs. 2.2%, *P* = 0.085) was significantly higher in the exposed group (Table 1).

**Table 1:** Comparison of clinical characteristics between the exposed group with CMV disease and the matched unexposed group without CMV diseases

Characteristics	CMV diseases		*P*-value
	No	Yes	
	(*n* = 3435)	(*n* = 687)	
Sex, male	1950 (56.77)	390 (56.77)	> 0.999
Age, years	57.98 ± 11.41	57.98 ± 11.42	> 0.999
≥ 60 years	1405 (40.9)	281 (40.9)	> 0.999
Duration of total follow-up, years	4.10 ± 1.58	3.73 ± 1.80	< 0.001
Low income status^a^	824 (23.99)	181 (26.35)	0.189
BMI, kg/m^2^	23.39 ± 5.95	22.63 ± 6.72	0.318
≥ 25 kg/m^2^	832 (24.22)	163 (23.73)	0.294
Co-morbid diseases, yes^b^	192 (5.59)	258 (37.55)	< 0.001
Solid organ transplant recipients	30 (0.87)	146 (21.25)	< 0.001
Kidney	27 (0.79)	109 (15.87)	< 0.001
Liver	3 (0.09)	19 (2.77)	< 0.001
Heart	0 (0)	13 (1.89)	–
Lung	0 (0)	5 (0.73)	–
HSCT recipients	16 (0.47)	95 (13.83)	< 0.001
Malignant neoplasms^c^	113 (3.29)	69 (10.04)	< 0.001
End-stage renal disease on dialysis	92 (2.68)	35 (5.09)	< 0.001
NIDDM	386 (11.24)	205 (29.84)	< 0.001
Hypertension	974 (28.36)	328 (47.74)	< 0.001
Dyslipidaemia	607 (17.67)	201 (29.26)	< 0.001
Moderate-to-severe dementia			
All	105 (3.06)	38 (5.53)	0.001
Alzheimer’s	77 (2.24)	23 (3.35)	0.085
Vascular	12 (0.35)	7 (1.02)	0.018
Other	16 (0.47)	8 (1.16)	0.028

Data are expressed as number (percentage) and median ± standard deviation

*Abbreviations*: *BMI* body mass index, *CMV* cytomegalovirus, *ESRD* end-stage renal disease, *HSCT* haematopoietic stem cell transplantation, *NIDDM* non-insulin-dependent diabetes mellitus, *SOT* solid organ transplantation

^a^Lower 25th percentile of socioeconomic status

^b^Patients with ≥ one among co-morbid diseases of SOT recipients, HSCT recipients, malignant neoplasms, and ESRD on dialysis

^c^Including haematologic malignancies and excluding in situ neoplasms

### Effect of CMV diseases on moderate-to-severe dementia development

The exposed group (14.8/1000 person-years) had two-fold higher IRs for all types of dementia than the unexposed group (7.5/1000 person-years). In M1, the odds ratio (OR) was 2.0 (95% confidence intervals [CI], 1.4–2.9), in M2 the OR was 2.1 (95% CI, 1.4–3.0), and in M3 the OR was 1.9 (95% CI, 1.2–2.8). In subgroup analyses based on dementia type, the IR of AD in the exposed group (9.0/1000 person-years) was 1.6 times higher than that of the unexposed group (5.5/1000 person-years), with an OR of 1.6 (95% CI, 1.0–2.6) in M3. The IR of VaD in the exposed group (2.7/1000 person-years) was up to three-fold higher than the unexposed group (0.9/1000 person-years), with an OR of 2.9 (95% CI, 1.1–7.5) in M3. The IR of other dementia except AD and VaD in the exposed group (3.1/1000 person-years) was 2.7 times higher than that in the unexposed group (1.1/1000 person-years) (Table 2).

**Table 2:** Multivariate logistic regression analyses to examine the effect of cytomegalovirus diseases on the development of moderate-to-severe dementia

Type of dementia	CMV diseases	Number of participants	Events	Total follow-up duration (person-years)	IR^a^	OR (95% CI)		
						Model 1	Model 2	Model 3
**All**	No	3435	105	14,083	7.46	1 (Ref.)	1 (Ref.)	1 (Ref.)
	Yes	687	38	2564	14.82	1.99 (1.37–2.88)	2.07 (1.42–3.03)	1.88 (1.23–2.82)
**AD**	No	3435	77	14,083	5.47	1 (Ref.)	1 (Ref.)	1 (Ref.)
	Yes	687	23	2564	8.97	1.65 (1.04–2.63)	1.69 (1.04–2.75)	1.62 (1.01–2.64)
**VaD**	No	3435	12	14,083	0.85	1 (Ref.)	1 (Ref.)	1 (Ref.)
	Yes	687	7	2564	2.73	3.14 (1.24–7.98)	3.30 (1.28–8.40)	2.87 (1.08–7.45)
**Others**	No	3435	16	14,083	1.14	1 (Ref.)	1 (Ref.)	1 (Ref.)
	Yes	687	8	2564	3.12	2.71 (1.16–6.34)	2.81 (1.89–6.65)	2.69 (1.07–6.43)

Data are expressed as number or OR (95% CI). Events indicate the new-onset moderate-to-severe dementia. Model 1, non-adjusted; Model 2, age-, sex-, low income status, and BMI-adjusted; Model 3, age-, sex-, low income status-, BMI-, SOT recipients-, HSCT recipients-, malignant neoplasms-, ESRD on dialysis-, NIDDM-, hypertension-, and dyslipidaemia-adjusted

*Abbreviations*: *AD* Alzheimer’s disease, *BMI* body mass index, *CI* confidence interval, *CMV* cytomegalovirus, *ESRD* end-stage renal disease, *HSCT* haematopoietic stem cell transplantation, *IR* incidence rate, *NIDDM* non-insulin-dependent diabetes mellitus, No., number of patients, *OR* odds ratio, *Ref.* reference, *SOT* solid organ transplantation, *VaD* vascular dementia

^a^Per 1000 person-years

### Incidence probability of new-onset moderate-to-severe dementia

**Fig. 1:**
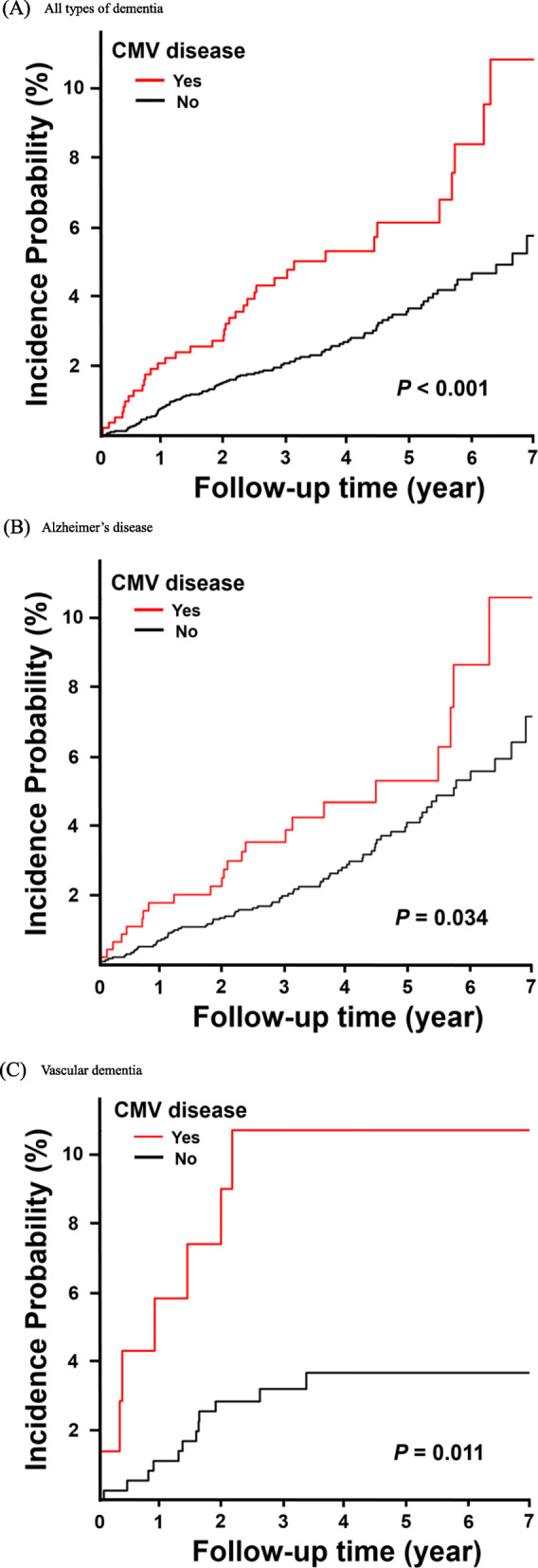
Kaplan-Meier curves for incidence probability of moderate-to-severe dementia in patients with cytomegalovirus (CMV) diseases (exposed group) and subjects without CMV diseases (unexposed group), adjusted by age, and sex. **a** All types of dementia. **b** Alzheimer’s disease. **c** Vascular dementia

During the 7-year follow-up period, the incidence probability of all types of dementia (*P* <  0.001) was significantly higher in the exposed group than that in the unexposed group (Fig. 1a). The incidence probabilities of AD (*P* = 0.034) and VaD (*P =* 0.011) were also significantly higher in the exposed group than the unexposed group (Fig. 1b, c). The slope of the Kaplan-Meier curves indicates that the incidence probability of AD was fairly different between groups, 5 years after follow-up initiation. By contrast, VaD mostly occurred within 3 years of CMV tissue-invasive end-organ disease diagnosis (Fig. 1b, c).

### Effect of CMV diseases on moderate-to-severe dementia according to sex and age

The IRs of all dementia types in the exposed and unexposed groups were not significantly different between men and women (OR in M3, 2.0 vs. 1.9, *P* = 0.648). The OR for IRs of all dementia types and AD in the two groups were significantly higher in subjects aged 40–59 years than in those aged 60–79 and ≥ 80 years (OR in M3, 11.7 vs. 1.8 vs. 1.3, *P* = 0.025 and OR in M3, 13.6 vs. 1.8 vs. 1.0, *P* = 0.037, respectively). In contrast, the IR of VaD was not significantly different among subjects aged 40–59, 60–79 and ≥ 60 years (*P* = 0.781) (Table 3).

**Table 3:** Multivariate logistic regression models to examine the effect of cytomegalovirus diseases on the development of moderate-to-severe dementia according to sex and age distribution

Type of dementia	Subgroups		CMV diseases	Number of participants	Events	IR^a^	OR (95% CI)			*P*-value
							Model 1	Model 2	Model 3	
**All**	**Sex**	**M**	No	1950	42	5.20	1 (Ref.)	1 (Ref.)	1 (Ref.)	0.648
			Yes	390	17	11.45	2.19 (1.25–3.85)	2.26 (1.27–3.99)	2.02 (1.09–3.59)	
		**F**	No	1485	63	10.50	1 (Ref.)	1 (Ref.)	1 (Ref.)	
			Yes	297	21	19.46	1.86 (1.13–3.04)	1.91 (1.09–3.12)	1.93 (1.14–3.23)	
	**Age**	**40–59**	No	2030	3	0.35	1 (Ref.)	1 (Ref.)	1 (Ref.)	0.025
			Yes	406	7	4.28	12.31 (3.18–47.60)	12.47 (3.14–48.76)	11.68 (2.45–49.37)	
		**60–79**	No	1260	62	12.47	1 (Ref.)	1 (Ref.)	1 (Ref.)	
			Yes	252	21	24.48	1.97 (1.20–3.24)	2.00 (1.21–3.34)	1.83 (1.07–3.15)	
		**≥ 80**	No	145	40	86.74	1 (Ref.)	1 (Ref.)	1 (Ref.)	
			Yes	29	10	145.71	1.62 (0.81–3.24)	1.38 (0.69–2.88)	1.31 (0.49–2.83)	
**AD**	**Sex**	**M**	No	1950	29	3.59	1 (Ref.)	1 (Ref.)	1 (Ref.)	0.197
			Yes	390	13	8.76	2.44 (1.27–4.69)	2.47 (1.25–4.72)	2.37 (1.21–4.62)	
		**F**	No	1485	48	8.00	1 (Ref.)	1 (Ref.)	1 (Ref.)	
			Yes	297	10	9.27	1.17 (0.59–2.31)	1.22 (0.60–2.45)	1.28 (0.61–2.59)	
	**Age**	**40–59**	No	2030	2	0.23	1 (Ref.)	1 (Ref.)	1 (Ref.)	0.037
			Yes	406	4	2.44	10.57 (1.94–57.69)	10.62 (1.88–58.76)	13.59 (2.14–86.26)	
		**60–79**	No	1260	46	9.25	1 (Ref.)	1 (Ref.)	1 (Ref.)	
			Yes	252	14	16.32	1.80 (0.99–3.27)	1.89 (1.01–3.45)	1.77 (0.99–3.30)	
		**≥ 80**	No	145	29	62.89	1 (Ref.)	1 (Ref.)	1 (Ref.)	
			Yes	29	5	72.85	1.14 (0.44–2.96)	1.02 (0.36–2.70)	1.01 (0.33–2.76)	
**VaD**	**Sex**	**M**	No	1950	7	0.87	1 (Ref.)	1 (Ref.)	1 (Ref.)	0.243
			Yes	390	2	1.35	1.52 (0.32–7.32)	1.58 (0.29–7.90)	1.27 (0.19–6.54)	
		**F**	No	1485	5	0.83	1 (Ref.)	1 (Ref.)	1 (Ref.)	
			Yes	297	5	4.63	5.47 (1.58–18.88)	5.42 (1.47–17.87)	4.31 (1.08–16.51)	
	**Age**	**40–59**	No	2030	1	0.12	1 (Ref.)	1 (Ref.)	1 (Ref.)	0.781
			Yes	406	1	0.61	5.21 (0.33–83.36)	5.24 (0.28–78.93)	1.18 (0.05–23.31)	
		**60–79**	No	1260	7	1.41	1 (Ref.)	1 (Ref.)	1 (Ref.)	
			Yes	252	3	3.50	2.40 (0.62–9.26)	2.44 (0.60–9.52)	1.83 (0.36–7.69)	
		**≥ 80**	No	145	4	8.67	1 (Ref.)	1 (Ref.)	1 (Ref.)	
			Yes	29	3	43.71	4.66 (1.04–20.90)	4.34 (0.82–17.59)	5.29 (0.78–31.74)	
**Others**	**Sex**	**M**	No	1950	6	0.74	1 (Ref.)	1 (Ref.)	1 (Ref.)	0.642
			Yes	390	2	1.35	1.80 (0.36–8.92)	1.82 (0.23–9.43)	1.56 (0.21–7.96)	
		**F**	No	1485	10	1.67	1 (Ref.)	1 (Ref.)	1 (Ref.)	
			Yes	297	6	5.56	3.28 (1.19–9.03)	3.15 (1.07–8.77)	3.15 (1.05–9.23)	
	**Age**	**40–59**	No	2030	0	0.0	–	–	–	–
			Yes	406	2	1.22	–	–	–	
		**60–79**	No	1260	9	1.81	1 (Ref.)	1 (Ref.)	1 (Ref.)	
			Yes	252	4	4.66	2.52 (0.77–8.17)	2.37 (0.65–8.37)	2.41 (0.68–8.29)	
		**≥ 80**	No	145	7	15.18	1 (Ref.)	1 (Ref.)	1 (Ref.)	
			Yes	29	2	29.14	1.74 (0.36–8.41)	1.19 (0.20–6.21)	1.31 (0.23–7.03)	

Data are expressed as number or OR (95% CI). Events indicate the new-onset moderate-to-severe dementia. Model 1, non-adjusted; Model 2, age-, sex-, low income status, and BMI-adjusted; Model 3, age-, sex-, low income status-, BMI-, SOT recipients-, HSCT recipients-, malignant neoplasms-, ESRD on dialysis-, NIDDM-, hypertension-, and dyslipidaemia-adjusted

*Abbreviations*; *AD* Alzheimer’s disease, *BMI* body mass index, *CI* confidence interval, *CMV* cytomegalovirus, *ESRD* end-stage renal disease, *F* female, *HSCT* haematopoietic stem cell transplantation, *IR* incidence rate, *M* male, *No* number of patients, *NIDDM* non-insulin-dependent diabetes mellitus, *OR* odds ratio, *Ref.* reference, *SOT* solid organ transplantation; *VaD* vascular dementia

^a^Per 1000 person-years

## Discussion

This study showed that the new-onset incidence of all types of moderate-to-severe dementia and each type of AD or VaD were significantly higher in subjects with CMV tissue-invasive end-organ diseases. After adjusting for age, sex, low income status, BMI, NIDDM, hypertension, and dyslipidaemia as well as the immunocompromised co-morbid diseases which can be risk factors for dementia [1–3, 25], patients diagnosed with CMV diseases had a higher risk for VaD than for AD (OR, 2.9 vs. 1.6). Previous studies have suggested that the chronic infection of murine CMV (MCMV) in blood vessels could cause atherosclerotic plaque progression, which could subsequently induce VaD via atherosclerosis pathogenesis in mouse models [26]. However, it is difficult to simulate the pathogenesis of human CMV (HCMV) diseases in a MCMV mouse model, because HCMV and MCMV are genetically different and mice cannot be infected with HCMV. Although the relevant infectious agents of VaD have not been studied more than those of AD, the stronger association of CMV diseases with VaD than AD is an interesting finding of this study. This result suggests that CMV may play a role in complex VaD pathogenesis, as active recurrent intermittent CMV replication is associated with chronic inflammatory atherosclerosis, vascular damage, and ultimately, cerebrovascular/cardiovascular diseases (CVD) [7, 27, 28].

Interestingly, our data showed that the IR of AD in the exposed group was higher in those aged 40–59 years than in those aged ≥60 years. The possible pathogeneses or causes between CMV diseases and moderate-to-severe AD, particularly early-onset AD, have not yet been studied. Generally, persistent systemic infections and inflammation can affect chronic neurodegeneration [29]. In particular, decreased CMV–specific memory CD8+ T lymphocyte counts and resulting cognitive dysfunction is one feature of AD [30]. One study revealed that cognitive impairment was six times higher in patients with the risk factors of low educational level, apolipoprotein E (APOE) ε4 allele, and *Herpesviridae,* including CMV, infection [31].

The ε4 allele of APOE, a genetic risk factor for AD and CVDs, has been shown to induce oxidative damage in the central nervous system in herpes simplex virus (HSV) infections [32]. Neurotoxicity and neuroinflammation by APOE ultimately result in neurodegeneration and dementia [4, 32, 33]. When APOE ε4 is expressed, β-amyloid aggregates in the brain, and amyloid plaques can cause cerebral amyloid angiopathy [34]. Moreover, the APOE ε4 allele is associated with new-onset AD or a decline in cognition in early clinical stages [35–39]. However, our cohort did not include data on APOE genotype status, due to the registry-based clinical data collection. Therefore, future studies would benefit from evaluating the APOE genotype in dementia patients with CMV tissue-invasive diseases.

Since VaD and AD have different pathogeneses and risk factors, their time of onset may differ. According to the Kaplan-Meier curves adjusted by age and sex, after a diagnosis of CMV diseases AD occurs at a relatively later clinical stage than VD, in spite of the high risk of early-onset at a younger age. The possible pathogenesis of AD arising from the deposition of amyloid plaques as a result of chronic inflammation after active CMV replication could explain why AD occurs at a later clinical stage [34]. Additionally, VaD group could have a tendency to drop out earlier during the following period because of their potential uneven life expectancy or systemic diseases including complication of CVD, as compared with AD group.

The majority (634 of 687 patients, 92.3%) of patients in the exposed group had the ICD-10 code B25.8 (other cytomegaloviral disease) or B25.9 (cytomegaloviral disease, unspecified). This finding may indicate that the CMV-infected organs were mainly those of the gastrointestinal tract including the oesophagus, stomach, colon, and rectum, and is supported by another study using CMV-infected formalin-fixed paraffin-embedded tissue in our hospital [40]. We did not find that the differential risk of moderate-to-severe dementia varied by CMV-infected organs. Recently, a meta-analysis with four case-control studies by Charlotte et al reported that CMV DNA in the brain was not associated with dementia including mild cognitive impairment (MCI) [12]. The divergent results from our study may be due to different definitions or severity of dementia, as well as race. In addition, it is possible that there is a pathogenic role of systemic inflammation caused by active CMV replication, rather than direct invasion of CMV into the brain.

This study has the potential biases and limitations associated with registry-based data. In addition, although we performed multivariate regression analyses adjusted for several co-morbid medical diseases that could impact cognitive function, there could have been a bias in the heterogeneously exposed population that may be present in neurobehavioral disturbances mimicking dementia (e.g. long-term metabolic encephalopathy in the immunocompromised patients and non-immunocompromised critically ill patients). For both groups, our large, population-based cohort study did not allow precise determination of patients’ cognitive level at initial enrolment or gradual cognitive decline, using various clinical staging scale measurements to detect MCI prior to moderate-to-severe dementia diagnosis during follow-up. Therefore, we were unable to evaluate an association of MCI with CMV end-organ diseases in this study. Because the diagnosis of moderate-to-severe dementia and prescription of anti-dementia drugs were simultaneously regulated by the Korean National Health Insurance Corporation for claiming NHIS insurance benefits, it may have been impossible for false-positive cases indicating spontaneous improvement of moderate-to-severe dementia without medical treatment to occur in this study. The patients at risk for CMV reactivation may also have experienced reactivation of other herpes viruses, particularly HSV-1 and 2, which have been evaluated as one risk factor for AD [12, 41]. However, we could not exclude or adjust for HSV infections, because many cases of other viral diseases including HSV could have been underdiagnosed due to self-limited disease course, and consequently would have missed a precise ICD-10 code. In addition, we could not analyse several markers of inflammation (interferon-gamma, tumour necrosis factor-alpha, and interleukin-6, etc.). The incidence or causes of dementia may differ by race; however, we were only able to investigate characteristics of dementia in Korean subjects. Finally, although income status was included in our cohort, the population-based dataset made it impossible to obtain the education level of each subject.

However, this study also had several strengths. First, only patients diagnosed with tissue-invasive end-organ diseases were included in the exposed group, regardless of CMV serostatus. This is a major distinctive characteristic of this study. The serum CMV IgG titre can vary at different time-points in each individual, and a positive/negative result only represents latent status caused by past CMV infection, rather than representing active CMV replication. Second, we defined dementia with moderate-to-severe severity and excluded MCI cases to maximally decrease any ambiguousness in dementia diagnosis and false positive cases. This could mean that our cohort appropriately assessed cognitive impairment that was clinically serious enough to interfere with daily activities. Third, we employed a nationwide population-based study using a huge database to analyse the effect of CMV on new-onset moderate-to-severe dementia after a CMV diagnosis through the washout period. In addition, previous studies did not include the time of dementia occurrence during follow-up. However, we analysed the onset time for dementia after the diagnosis of CMV diseases for a relatively long duration of 7 years. Fourth, we excluded HIV-1-infected individuals from the cohort, which could remove HIV-associated neurocognitive disorders and/or HIV-associated dementia caused by HIV-1 infection itself.

## Conclusion

Patients with CMV tissue-invasive end-organ diseases had a higher risk of moderate-to-severe dementia than those without CMV diseases. Even though the causes of dementia are multifactorial and may be genetic, educational, and environmental in nature, CMV end-organ diseases may be associated with the development of moderate-to-severe dementia, particularly VaD or AD, in individuals aged 40–59 years.

## Supplementary information

**Additional file 1: Table S1**. Disease-related categories and the linked ICD-10 and unique codes for rare intractable diseases for Alzheimer’s disease, vascular dementia, and other dementia used in this cohort.

## Data Availability

The authors are not allowed to share the analysis datasets of the current study due to data regulations.

## Abbreviations

AD: Alzheimer’s disease
APOE: Apolipoprotein E
BMI: Body mass index
CI: Confidence interval
CMV: Cytomegalovirus
CVD: Cerebrovascular/cardiovascular diseases
ESRD: End-stage renal disease
HCMV: Human cytomegalovirus
HIRA: The Korean Health Insurance Review and Assessment
ICD: The International Statistical Classification of Diseases
HIV: Human immunodeficiency virus
HSCT: Haematopoietic stem cell transplantation
HSV: Herpes simplex virus
IgG: Immunoglobulin G
IR: Incidence rate
MCI: Mild cognitive impairment
MCMV: Murine cytomegalovirus
NHID: National Health Insurance Database
NHIS: National Health Insurance Service
NIDDM: Type 2 non-insulin-dependent diabetes mellitus
OR: Odds ratio
RID: Rare incurable disease
SOT: Solid organ transplantation
VaD: Vascular dementia
