# Supplementary material for: Association between cytomegalovirus end-organ diseases and moderate-to-severe dementia: a population-based cohort study
Source: BMC Neurol. 2020 May 28;20:216. doi: 10.1186/s12883-020-01776-3 (PMC7254693; doi:10.1186/s12883-020-01776-3)
Supplement: Supplementary file 1 — Additional file 1: Table S1. Disease-related categories and the linked ICD-10 and unique codes for rare intractable diseases for Alzheimer’s disease, vascular dementia, and other dementia used in this cohort. [file 12883_2020_1776_MOESM1_ESM.docx]

**Supplementary Table 1**. Disease-related categories and the linked ICD-10 and unique codes for rare intractable diseases for Alzheimer’s disease, vascular dementia, and other dementia used in this cohort

| **RID codes^a^** | **ICD-10 codes^b^** | **Diseases** |
| --- | --- | --- |
| V800 | F00.0/G30.0 | 1. Dementia in AD with early onset |
|  |  | 2. AD, type 2 |
|  |  | 3. Presenile dementia, Alzheimer’s type |
|  |  | 4. Primary degenerative dementia of the Alzheimer’s type, presenile onset |
|  | F02.0/G31.0 | Dementia in Pick disease |
|  | F02.1/A81.0 | Dementia in Creutzfeldt-Jakob disease |
|  | F02.2/G10 | Dementia in Huntington disease |
|  | F02.3/G20 | Dementia in Parkinson disease |
|  | F02.8/G31.8 | Dementia with Lewy bodies disease |
|  | G31.0 | Frontotemporal dementia |
| V810 | F00.1/G30.1 | 1. Dementia in AD with late onset |
|  |  | 2. AD, type 1 |
|  |  | 3. Primary degenerative dementia of the Alzheimer’s type, senile onset |
|  |  | 4. Senile dementia, Alzheimer’s type |
|  | F00.2/G30.8 | 1. Dementia in AD, atypical or mixed type |
|  |  | 2. Atypical dementia, Alzheimer type |
|  | F00.9/G30.9 | Dementia in Alzheimer disease, unspecified |
|  | F01.0 | Vascular dementia of acute onset |
|  | F01.1 | 1. Multi-infarct dementia |
|  |  | 2. Predominantly cortical dementia |
|  | F01.2 | Subcortical vascular dementia |
|  | F01.3 | Mixed cortical and subcortical vascular dementia |
|  | F01.8 | Other vascular dementia |
|  | F01.9 | Vascular dementia, unspecified |

^a^Presented by the Korean National Insurance Service, ^b^Presented by the World Health Organization. Aberrations; AD, Alzheimer’s disease; ICD-10, the International Statistical Classification of Diseases and Related Health Problems 10th Revision; RID, rare intractable disease.
